# Nuclear Respiratory Factor-1 Ameliorates Heart Failure by Suppressing Cardiomyocyte Pyroptosis-Associated Signaling Via the Downregulation of Gasdermin D and Caspase-1

**DOI:** 10.14740/cr2153

**Published:** 2026-04-15

**Authors:** Fei Dong, Cai Xia Zhang, Guang Tao Zhang, Yan Yang, Jun Ling Pan, Jian Xiang Lu, Yin Li Luo, Xia Li

**Affiliations:** a The Shizuishan Second People’s Hospital, Shizuishan, Ningxia Hui Autonomous Region 753000, China; b These authors contributed equally to this work.

**Keywords:** Heart failure, Pyroptosis, NRF-1, GSDMD, Caspase-1

## Abstract

**Background:**

Cardiac diseases caused by various factors eventually lead to heart failure (HF) as the condition progresses, during which inflammation and pyroptosis are markedly enhanced. Nuclear respiratory factor-1 (NRF-1) is a transcriptional regulator involved in multiple physiological functions; however, its role in pyroptosis during HF remains unclear.

**Methods:**

Serum samples from patients with HF were collected to evaluate the levels of NRF-1. An HF rat model was established to assess the expression of NRF-1 in serum and cardiac tissue and to investigate its association with HF and the expression of inflammatory markers gasdermin D (GSDMD), caspase-1, interleukin (IL)-8, and IL-1β. NRF-1-overexpressing and NRF-1-silenced H9C2 cell lines were constructed, and myocardial injury was induced by hypoxia and doxorubicin (DOX) to evaluate the effects of NRF-1 on pyroptosis-related molecules GSDMD and caspase-1, as well as inflammatory cytokines IL-8 and IL-1β. Finally, the expression of NRF-1 in the serum of HF patients was analyzed based on New York Heart Association (NYHA) functional classification to validate the dynamic changes of NRF-1 during pyroptosis in HF.

**Results:**

Although previous studies have reported inconsistent findings regarding serum NRF-1 expression levels among different HF patient cohorts, our current results demonstrate that serum NRF-1 expression is significantly reduced in HF patients compared to those with normal cardiac function (NF), while the expression of pyroptosis-related molecules GSDMD and caspase-1, as well as pro-inflammatory cytokines IL-8 and IL-1β, is markedly increased. These findings were further validated in an HF rat model. *In vitro* experiments revealed that NRF-1 attenuates hypoxia and DOX-induced pyroptosis in H9C2 cardiomyocytes, highlighting its protective role in the pathogenesis of HF. Finally, serum NRF-1 levels assessed according to NYHA functional classification suggest that the differential expression of NRF-1 observed across samples may be attributed to variations in the stages of HF among patients.

**Conclusions:**

NRF-1 is a dynamically expressed molecule with cardioprotective properties that ameliorates HF and attenuates pyroptosis by inhibiting the caspase-1/GSDMD signaling pathway.

## Introduction

Heart failure (HF) is a progressive clinical syndrome characterized by structural and/or functional abnormalities of the myocardium caused by multiple factors, representing the end stage of many cardiovascular diseases and typically accompanied by elevated levels of natriuretic peptides [1, 2]. As a global health issue, HF is associated with high morbidity and mortality, poor quality of life, and limited therapeutic options, affecting over 64 million people worldwide [3]. Due to frequent hospitalizations and associated healthcare expenditures, HF imposes a substantial socioeconomic burden [4].

Pyroptosis is a recently identified form of programmed cell death that is distinct from both necrosis and apoptosis and is characterized by the release of pro-inflammatory cytokines [5]. This process involves the activation of inflammasomes, caspase family proteins, the gasdermin (GSDM) family, and inflammatory mediators such as interleukin (IL)-18) and IL-1β [6]. Pyroptotic cells typically undergo swelling, followed by membrane rupture, leading to the release of cytoplasmic contents into the extracellular space. These released components can be recognized by neighboring cells, thereby triggering inflammatory responses [7]. Inflammation plays a pivotal role in HF [8–10]. During the progression of HF, cardiomyocytes are subjected to persistent stressors such as pressure overload and hypoxia, which can induce pyroptosis [11]. The inflammation elicited by pyroptosis further damages cardiomyocytes, impairs tissue repair, and exacerbates HF, thereby creating a vicious cycle [12–14].

The nuclear respiratory factor-1 (NRF-1) is a key nuclear transcriptional regulator that controls mitochondrial biogenesis [15]. By coordinating gene expression between the nuclear and mitochondrial genomes, NRF-1 plays a critical regulatory role in oxidative phosphorylation, heme biosynthesis, and the transcription and replication of DNA [16–19]. In addition to its role in mitochondrial function, NRF-1 is involved in the regulation of various disease processes and is a crucial mediator of multiple gene expression pathways [20]. Some studies have shown that NRF-1 can protect H9C2 cells from hypoxia-induced apoptosis [21–23]. However, its role in HF, particularly in the context of pyroptosis, remains poorly understood.

Therefore, in this study, we investigated the role of NRF-1 in HF and examined its regulatory effect on pyroptosis. Our findings revealed that NRF-1 expression is dynamic and that it alleviates HF by inhibiting pyroptosis-associated signaling.

## Materials and Methods

### Animals and surgical protocols

Male Sprague-Dawley (SD) rats weighing approximately 220 ± 10 g were used in this study, which was approved by the Ethics Committee of the Second People’s Hospital of Shizuishan. The rats were housed under standard conditions with appropriate humidity (55±5%), temperature (23 ± 2 °C), and a 12-h light/dark cycle for 1 week before starting the experiment. Rats were weighed and anesthetized by intraperitoneal injection of 1% pentobarbital sodium at a dose of 50 mg/kg. Animals were randomly divided into three groups, with six rats in each group. Rats in the control (Ctrl) group received no treatment; the sham group underwent a sham operation, while the HF group underwent left anterior descending (LAD) coronary artery ligation to induce HF. Postoperatively, rats were monitored daily for clinical condition and body weight. After 4 weeks, serum and heart tissues were collected for subsequent analyses.

### Echocardiography

Cardiac assessments were performed using the Mindray M5 portable color Doppler ultrasound system (Shenzhen, China) equipped with a 3.5–4.0 MHz probe. During the examination, subjects were placed in the supine position. Initially, M-mode imaging was used to scan the apical four-chamber and left ventricular long-axis views.

### Enzyme-linked immunosorbent assay (ELISA)

For blood samples, the collected blood was collected into standard vacutainer tubes without anticoagulant. The samples were allowed to clot at room temperature for 30 min and then centrifuged at 3,000 rpm for 10 min at 4 °C. The resulting serum was carefully inspected, and samples with visible hemolysis were excluded. The supernatant was collected, aliquoted, and either subjected to immediate analysis or stored at –80 °C until use. For cell culture supernatants, the medium was aspirated and centrifuged at 3,000 rpm for 10 min at 4 °C. The clarified supernatant was collected, aliquoted, and used for subsequent assays or stored at –80 °C. ELISA kits were used to detect N-terminal pro-B-type natriuretic peptide (NT-proBNP, EK1393, Multi Science, Hangzhou, China), NRF-1 (E15022h, EIAab, Wuhan, China), IL-18 (EK118S, Multi Science, Hangzhou, China), IL-1β (EK101B, Multi Science, Hangzhou, China), gasdermin D (GSDMD) (E15480h, EIAab, Wuhan, China), and caspase-1 (E1592h, EIAab, Wuhan, China), following the instructions of manufacturers.

### Cell counting kit-8 (CCK-8) assay

When H9C2 cells reached approximately 90% confluence, they were detached from the culture dish using 0.25% trypsin. After cell counting, they were seeded into 96-well plates. Cells were then subjected to normoxic or hypoxic conditions in a time-staggered manner (from longest to shortest duration), ensuring that all samples were harvested simultaneously. Cell viability was subsequently assessed using the CCK-8 assay kit (40203ES60, YEASEN, Shanghai, China) according to the manufacturer’s instructions.

### Cell culture

H9C2 cells were obtained from the Chinese Academy of Sciences (Shanghai, China). NRF-1 overexpression (pCDH-NRF1) and NRF-1 knockdown (sh-NRF1) cell lines, constructed via lentiviral transduction, were retrieved from laboratory cryopreservation stocks [24]. Following cell thawing, the cells were cultured in complete medium under an atmosphere of 5% CO2. Once the cells reached optimal condition, total RNA was extracted to verify NRF-1 expression, ensuring the quality and suitability of the cell lines for subsequent experiments. For doxorubicin (DOX)-induced H9C2 cell injury, cells were treated with 1 µM DOX (23214-92-8, MCE, USA) for 24 h.

### Flow cytometry

H9C2 cells were seeded into six-well plates at a density of approximately 1 × 10^6^ cells per well and cultured in complete medium containing 10% fetal bovine serum (FBS) until reaching 70–80% confluence. Cells were then transferred to a hypoxia incubator (1% O_2_, 5% CO_2_, 94% N_2_) for 24 h to induce hypoxic stress. After treatment, the culture vessels were removed and allowed to equilibrate at room temperature. The supernatant was gently aspirated, and cells were washed twice with pre-chilled phosphate buffered saline (PBS), then resuspended at a concentration of 1 × 10^6^ cells/mL. To assess pyroptosis, detection was performed strictly according to the manufacturer’s instructions for the FAM-FLICA Caspase Assay Kit (ImmunoChemistry Technologies, Bloomington, MN, USA).

### Quantitative real-time polymerase chain reaction (qPCR)

Total RNA was extracted from treated H9C2 cells using TRIzol reagent (15596026CN, USA). Following quantification of RNA concentration, reverse transcription was performed to synthesize cDNA according to the instructions provided with the RevertAid First Strand cDNA Synthesis Kit (K1622, Invitrogen). qPCR was conducted using the BeyoFast™ SYBR Green qPCR Mix (D7262, Beyotime, Shanghai, China) following the manufacturer’s protocol. Relative gene expression was calculated using the 2–ΔΔCt method, with glyceraldehyde-3-phosphate dehydrogenase (GAPDH) serving as the internal reference gene. The primer sequences of the relevant genes are presented in Table 1.

**Table 1:** The Primer Sequences of the Relevant Genes

Gene	Primer sequence (5'–3')
*NRF1*	F: TCTGCTGTGGCTGATGGAGAGG
	R: GATGCTTGCGTCGTCTGGATGG
*GSDMD*	F: ATGAGGTGCCTCCACAACTTCC
	R: CCAGTTCCTTGGAGATGGTCTC
*CASP1*	F: GACCGAGTGGTTCCCTCAAG
	R: GACGTGTACGAGTGGGTGTT
*GAPDH*	F: ATGGCACAGTCAAGGCTGAGA
	R: CGCTCCTGGAAGATGGTGAT

F: forward primer; R: reverse primer.

### Western blot

Rat heart tissues and H9C2 cells were lysed using pre-chilled radioimmunoprecipitation assay (RIPA) lysis buffer (G2002, Servicebio, Wuhan, China). Cardiac tissue blocks were subjected to ultrasonic disruption on ice until fully lysed. The lysates were then centrifuged at 12,000 × g for 10–15 min at 4 °C, and the supernatants were collected as total protein extracts. Protein concentrations were determined using the bicinchoninic acid (BCA) protein assay kit (G2026, Servicebio, Wuhan, China), following the manufacturer’s instructions and calculating concentrations based on a standard curve. Equal amounts of protein were mixed with sodium dodecyl sulfate (SDS) loading buffer (G2075, Servicebio, Wuhan, China) and denatured at 95 °C for 5 min. Western blotting was performed following standard protocols, including electrophoresis and membrane transfer. After transfer, membranes were blocked with 5% non-fat milk in Tris buffered saline with Tween (TBST) at room temperature for 1 h to reduce nonspecific background, followed by incubation with primary antibodies overnight at 4 °C on a shaker. The next day, membranes were washed with TBST and incubated with horseradish peroxidase (HRP)-conjugated secondary antibodies at room temperature for 1 h. Protein bands were visualized using enhanced chemiluminescence (ECL) and captured with an imaging system. Band intensity was analyzed and quantified using ImageJ software. The primary antibodies used in this study were NRF-1 (ab175932, 1:2000, Abcam, UK), GSDMD (ab219800, 1:1000, Abcam, UK), caspase-1 (ab207802, 1:1000, Abcam, UK), IL-18 (ab243091, 1:500, Abcam, UK), IL-1β (ab283818, 1:500, Abcam, UK) and GAPDH (ab8245, 1:5000, Abcam, UK). The secondary antibody was HRP-conjugated anti-mouse immunoglobulin G (IgG) (ab6789, 1:500, Abcam, UK).

### Gene Expression Omnibus (GEO) data

All HF sample data used in this study were obtained from the GEO database. For microarray datasets, raw CEL files were processed with background correction and quantile normalization. For RNA sequencing (RNA-seq) datasets, raw counts were normalized using the voom function in the limma package. Differential expression analysis was performed using limma, and Benjamini–Hochberg false discovery rate (FDR) correction was applied to account for multiple comparisons. Differentially expressed genes (DEGs) were defined as |log_2_(fold change (FC))| > 1 with adjusted P < 0.05.

### Participants

A total of 45 participants were enrolled in this study, including 30 patients with HF and 15 age- and sex-matched healthy controls. Among the HF patients, 15 were classified as New York Heart Association (NYHA) functional class I and 15 as class IV. The study recruitment period was from December 25, 2023, to May 30, 2025. The HF cohort consisted of 18 males and 12 females, with a mean age of 63.5 ± 8.2 years. The control group included eight males and seven females, with a mean age of 61.2 ± 7.5 years. Inclusion criteria for HF patients were: 1) diagnosis of chronic HF based on current clinical guidelines; 2) age between 40 and 75 years; and (3) stable medical therapy for at least 4 weeks prior to enrollment. Exclusion criteria were: 1) acute decompensated HF or recent hospitalization (< 4 weeks); 2) history of malignancy, autoimmune disease, or severe hepatic/renal dysfunction; 3) active infection; and 4) inability to provide informed consent. Healthy controls were recruited from the same geographic region and were free of cardiovascular, metabolic, or systemic diseases.

### Ethics approval and consent to participate

The clinical samples involved in this study were approved by the Ethics Committee of the Second People's Hospital of Shizuishan (Approval No. 20231023-1), and the study was conducted in strict accordance with the Declaration of Helsinki and its subsequent amendments. Written informed consent was obtained from all participants prior to the commencement of the study. For illiterate participants, consent was obtained orally in the presence of an impartial witness and documented by the study staff. All human samples and related data collected during the research were anonymized to ensure participant privacy and data security.

All animal experiments in this study were approved by the Ethics Committee of the Second People’s Hospital of Shizuishan (Approval No. 2024080101) and were conducted in strict accordance with the guidelines of the Institutional Animal Care and Use Committee (IACUC) and the Guide for the Care and Use of Laboratory Animals. Appropriate anesthesia and euthanasia procedures were employed during the experimental process to minimize animal suffering.

### Statistical analysis

All experiments were conducted using randomization and blinded analysis to enhance the reliability of the conclusions. Data are presented as mean ± standard deviation (SD). For comparisons between two groups, Student’s *t*-test was used, while one-way analysis of variance (ANOVA) was applied for multiple group comparisons. Statistical analyses and graphical outputs were performed using GraphPad Prism version 8.0. A P value of less than 0.05 was considered statistically significant.

## Results

### Increased rate of pyroptosis-associated signaling in HF patients

**Figure 1:**
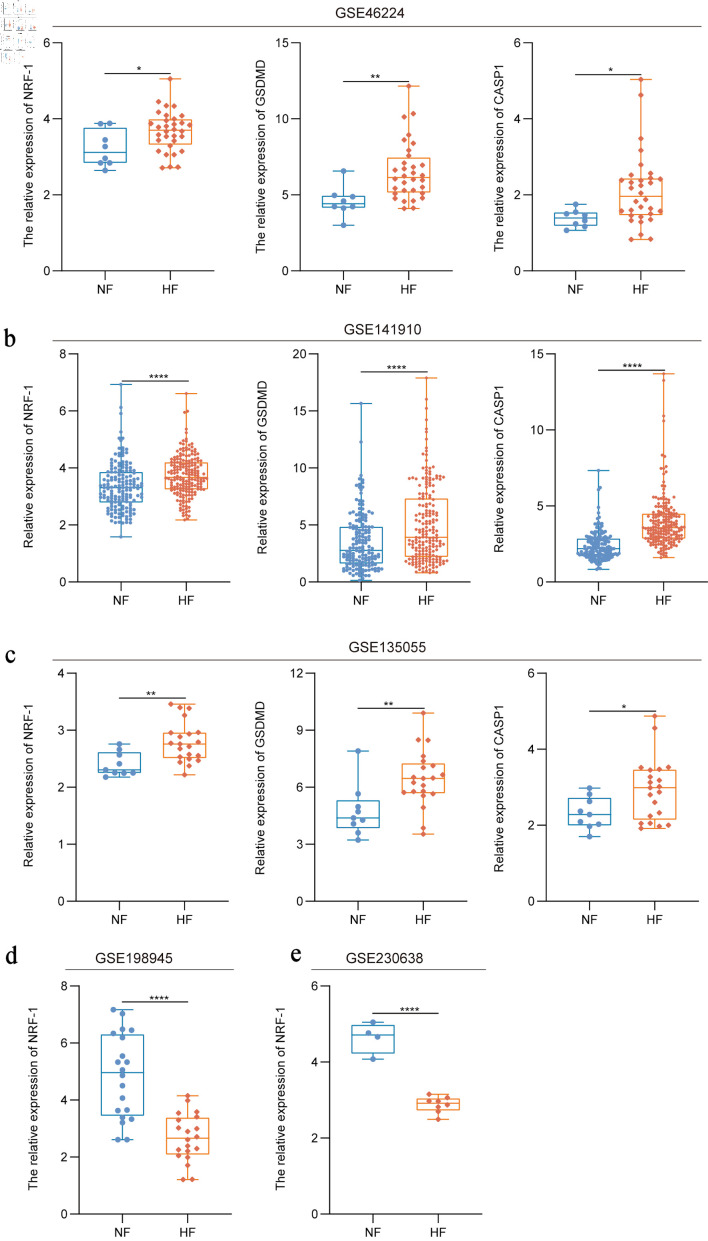
Increased rate of pyroptosis in HF patients. (a–c) Expression of NRF-1, GSDMD and CASP1 in HF patients compared to healthy controls in the GSE46224, GSE141910 and GSE135055 datasets. (d, e) Expression of NRF-1 in HF samples compared to healthy controls in the GSE198945 and GSE230638 datasets. *P < 0.05, **P < 0.01, ****P < 0.0001. CASP1: caspase 1; GSDMD: gasdermin D; HF: heart failure; NF: normal cardiac function; NRF-1: nuclear respiratory factor-1.

As a transcription factor, NRF is considered a critical protective factor in HF, with NRF-1 in particular known to ameliorate HF through its antioxidant, metabolic regulatory, and anti-inflammatory functions. Therefore, we obtained HF datasets from GSE46224, GSE 141910, and GSE 135055. Analysis revealed that, compared with NF individuals, the expression levels of NRF-1, GSDMD, and CASP1 were elevated in HF patients (P = 0.03/0.04, P = 0.007) (Fig. 1a–c), suggesting a significant increase in pyroptosis-associated signaling in HF patients. Interestingly, NRF-1 expression was found to be reduced in HF patients from two other datasets (GSE 198945 and GSE 230638) (P < 0.0001) (Fig. 1d, e).

### NRF-1 is negatively correlated with pyroptosis-associated signaling in HF patients

**Figure 2:**
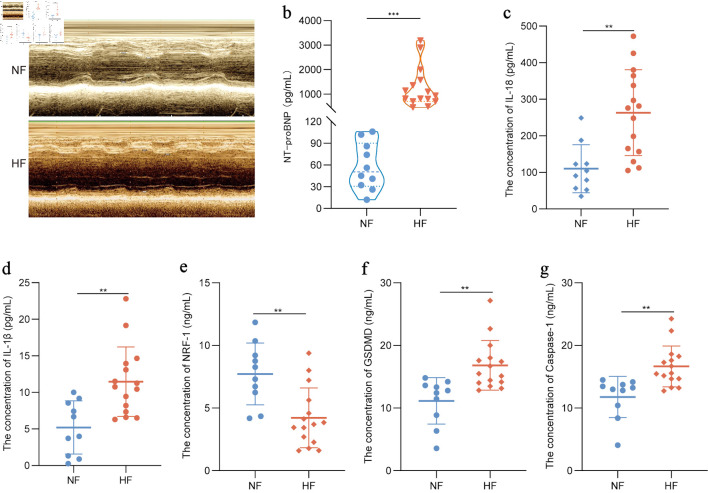
NRF-1 is negatively correlated with pyroptosis in HF patients. (a) Representative images of echocardiography in HF patients and NF controls. (b–g) Serum NT-proBNP, IL-18, IL-1β, NRF-1, GSDMD and caspase-1 level in HF patients (n = 15) and NF controls (n = 10). *P < 0.05, **P < 0.01, ***P < 0.001. GSDMD: gasdermin D; HF: heart failure; IL: interleukin; NF: normal cardiac function; NRF-1: nuclear respiratory factor-1; NT-proBNP: N-terminal pro-B-type natriuretic peptide.

NRF-1 exhibited differential expressions across various samples, presenting a compelling scientific question worthy of further investigation. To this end, we collected serum samples from 15 patients with HF for preliminary analysis (P = 0.0001) (Fig. 2a, b). In this study, compared with normal cardiac function (NF) individuals, HF patients exhibited elevated serum levels of the inflammatory cytokines IL-18 and IL-1β (P = 0.0011/0.0018) (Fig. 2c, d), along with increased expression of pyroptosis-related molecules GSDMD and caspase-1 (P = 0.0013/0.0015) (Fig. 2f, g), whereas the expression of NRF-1 was decreased (P = 0.0017) (Fig. 2e).

### NRF-1 is negatively correlated with pyroptosis-associated signaling in HF rats

**Figure 3:**
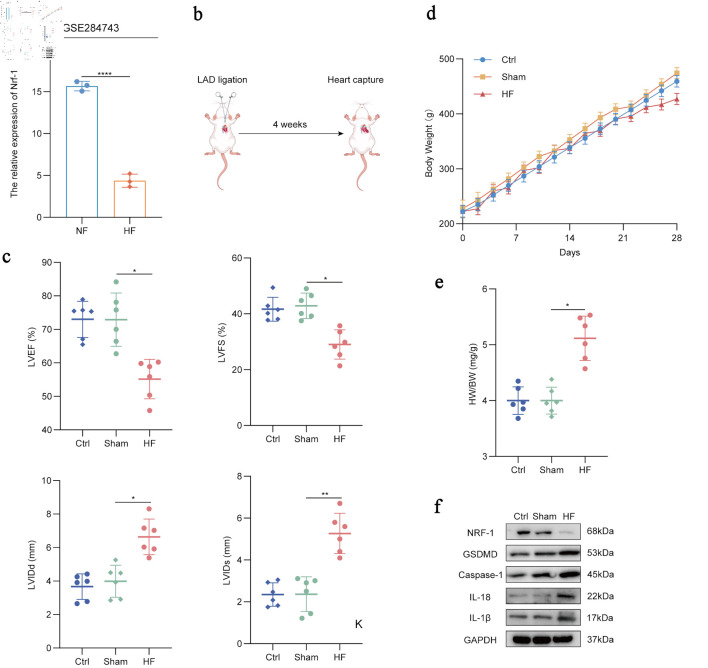
NRF-1 is negatively correlated with pyroptosis in HF rats. (a) Expression of Nrf-1 in HF samples compared to healthy controls in the GSE284743 datasets. (b) Schematic diagram of rat heart failure model. (c) Quantification of key ultrasound indicators including left ventricular ejection fraction (LVEF), left ventricular fractional shortening (LVFS), left ventricular internal dimension at end-diastole (LVIDd) and left ventricular internal dimension at end-systole (LVIDs) in HF rats (n = 6), sham rats (n = 6) and control rats (n = 6) for 4 weeks after successful modelling. (d) Body weight changes in HF rats (n = 6), sham rats (n = 6) and control rats (n = 6) 4 weeks after LAD ligation surgery. (e) Ratio of heart weight and body weight of HF rats (n = 6), sham rats (n = 6) and control rats (n = 6) at the fourth week after LAD ligation surgery. (f) Western blots of IL-18, IL-1β, NRF-1, GSDMD and caspase-1 level in HF rats (n = 6), sham rats (n = 6) and control rats (n = 6). *P < 0.05, **P < 0.01, ****P < 0.0001. Ctrl: control; HF: heart failure; IL: interleukin; LAD: left anterior descending; NF: normal cardiac function; NRF-1: nuclear respiratory factor-1; GAPDH: glyceraldehyde-3-phosphate dehydrogenase; GSDMD: gasdermin D; HW: heart weight; BW: body weight.

Results indicate that Nrf-1 expression is significantly reduced in HF model mice (P < 0.0001, GSE 284743) (Fig. 3a). To further explore this phenomenon of variation, we established a rat model of HF (Fig. 3b) and assessed cardiac function 4 weeks post-surgery. Compared with control rats, HF rats displayed increased left ventricular internal dimension at end-diastole (LVIDd) and left ventricular internal dimension at end-systole (LVIDs), along with reduced left ventricular ejection fraction (LVEF) and left ventricular fractional shortening (LVFS), indicating successful model establishment (P = 0.01/0.02, P = 0.002) (Fig. 3c). We also evaluated body weight and heart weight in the rats. The HF rats showed a slight but not statistically significant reduction in body weight, whereas the heart weight-to-body weight ratio (HW/BW) was significantly increased compared to controls (P = 0.01) (Fig. 3d, e). Subsequent analysis of protein expression revealed that, consistent with earlier findings, NRF-1 expression was downregulated in the HF model group, while the levels of pyroptosis and inflammation-related proteins, including GSDMD, caspase-1, IL-18, and IL-1β were elevated (Fig. 3f). These results suggest a negative correlation between NRF-1 expression and pyroptosis-associated signaling.

### NRF-1 inhibits H9C2 hypoxia-induced apoptosis and pyroptosis

**Figure 4:**
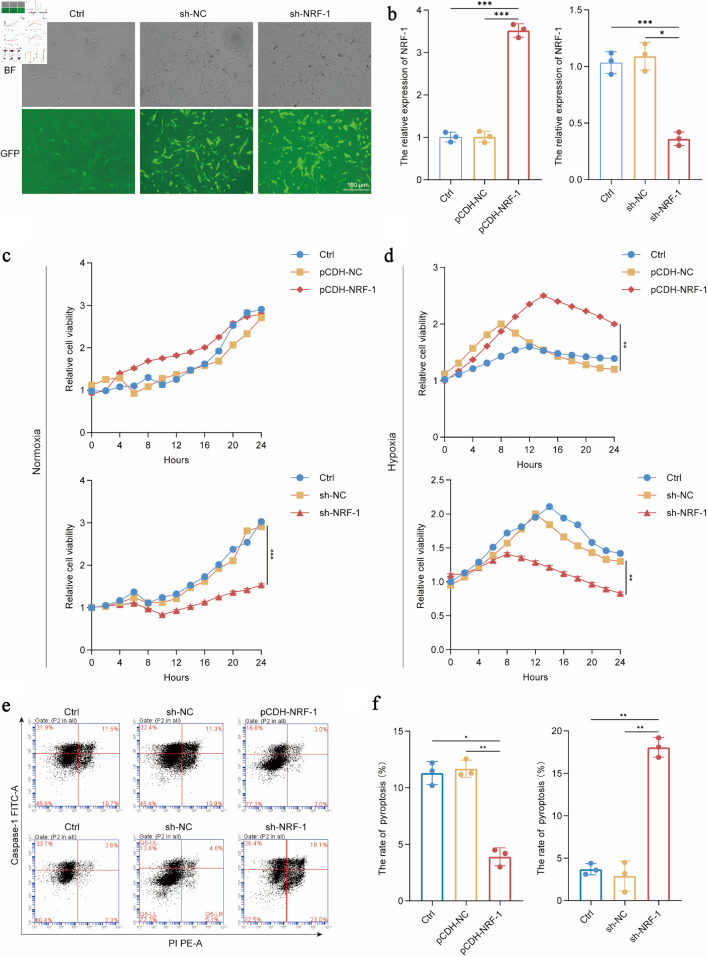
NRF-1 inhibits H9C2 hypoxia-induced apoptosis and pyroptosis. (a, b) Construction and validation of H9C2 overexpressing NRF-1 cell line (pCDH-NRF1) and inhibited cell line (sh-NRF-1) (× 200, scale bars = 100 µm). (c, d) Effects of NRF-1 on the proliferation of H9C2 cells under normoxia and hypoxia for 24 h. (e, f) Effects of NRF-1 on the pyroptosis of H9C2 cells under hypoxia for 24 h (n = 3). *P < 0.05, **P < 0.01, ***P < 0.001. NRF-1: nuclear respiratory factor-1; BF: bright field; GFP: green fluorescent protein; FITC: fluorescein isothiocyanate.

To further investigate the role of NRF-1 in pyroptosis, we established NRF-1 overexpression (pCDH-NRF1) and knockdown (sh-NRF1) cell lines in H9C2 cardiomyocytes [23]. After confirming the efficiency of NRF-1 modulation in these cell lines (P = 0.03, P = 0.0003/0.0009) (Fig. 4a, b), we examined the effect of NRF-1 on apoptosis under both normoxic and hypoxic conditions (24 h). The results showed that under normoxic conditions, NRF-1 overexpression did not affect cell viability, whereas NRF-1 knockdown induced apoptosis in H9C2 cells (P = 0.0005) (Fig. 4c). Under hypoxic conditions, H9C2 cells exhibited injury, and NRF-1 overexpression significantly enhanced cell viability, while NRF-1 knockdown promoted apoptosis (P = 0.006/0.008) (Fig. 4d). We then assessed the impact of NRF-1 on pyroptosis under hypoxia. Following 24 h of hypoxic exposure, NRF-1 overexpression markedly reduced the rate of pyroptosis, whereas NRF-1 knockdown significantly promoted pyroptosis in H9C2 cells (P = 0.02, P =0.0047/ 0.0092/0.0095) (Fig. 4e, f). These findings suggest that NRF-1 protects H9C2 cardiomyocytes from hypoxia-induced apoptosis and pyroptotic injury.

### NRF-1 inhibits the expression of pyroptosis core molecules

**Figure 5:**
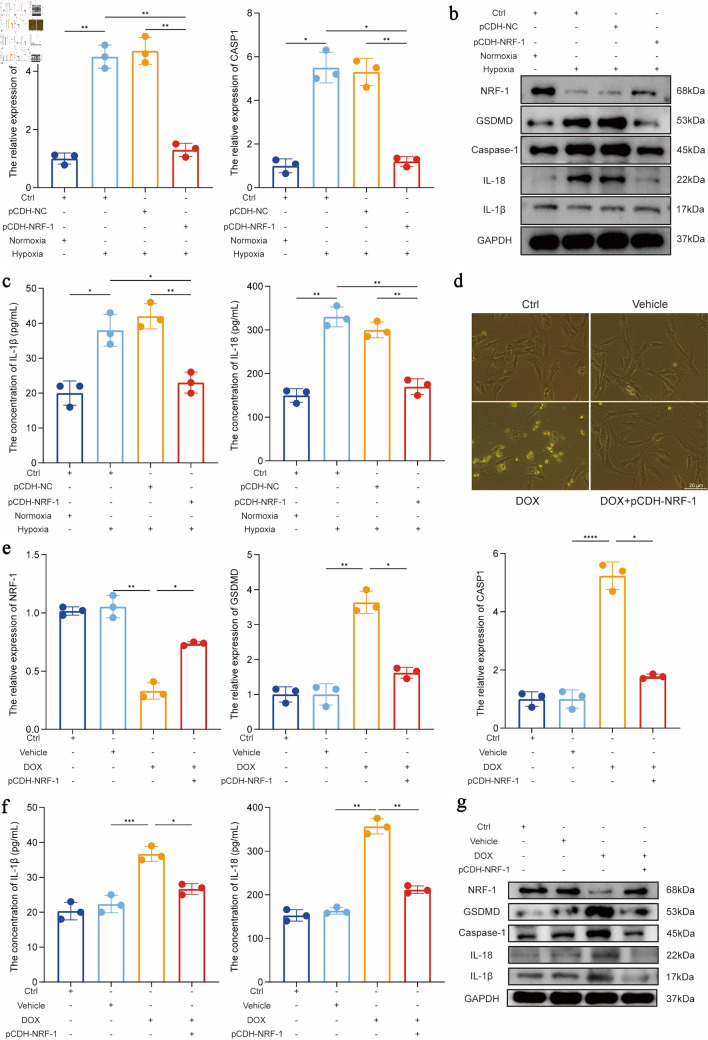
NRF-1 inhibits the expression of pyroptosis core molecules. (a) Effect of NRF-1 on RNA level expression of GSDMD and CASP1 under normoxia and hypoxia in H9C2. (b) Effect of NRF-1 on protein level expression of GSDMD, caspase-1, IL-18 and IL-1β under normoxia and hypoxia in H9C2. (c) Effect of NRF-1 on IL-18 and IL-1β expression in H9C2 cell culture supernatants under normoxia and hypoxia. (d) The figure presents Adriamycin (doxorubicin (DOX))-induced damage in H9C2 cells (× 400, scale bars = 20 µm). (e) Effect of NRF-1 on RNA level expression of GSDMD and CASP1 under DOX-induced H9C2. (f) Effect of NRF-1 on IL-18 and IL-1β expression in cell culture supernatants under DOX-induced H9C2. (g) Effect of NRF-1 on protein level expression of GSDMD, caspase-1, IL-18 and IL-1β under DOX-induced H9C2 (n = 3). *P < 0.05, **P < 0.01, ***P < 0.001. CASP1: caspase 1; GSDMD: gasdermin D; NRF-1: nuclear respiratory factor-1; Ctrl: control; GAPDH: glyceraldehyde-3-phosphate dehydrogenase.

Although flow cytometry results confirmed the protective effect of NRF-1 against pyroptosis in H9C2 cells under hypoxic conditions, we further investigated the molecular mechanisms of NRF-1 by assessing key pyroptosis-related molecules. After 24 h of hypoxia, NRF-1 overexpression significantly reduced the expression levels of GSDMD, caspase-1, IL-18, and IL-1β (P = 0.02/0.01, P = 0.001/0.004/0.005/0.007) (Fig. 5a–c). Additionally, we used DOX to induce cardiomyocyte injury in H9C2 cells to validate the role of NRF-1 in pyroptosis during HF. Microscopic images revealed that NRF-1 overexpression alleviated DOX-induced cellular damage (P = 0.02, P < 0.0001) (Fig. 5d). At the molecular level, NRF-1 overexpression reversed the DOX-induced downregulation of NRF-1 and attenuated the expression of key pyroptosis molecules, including GSDMD, caspase-1, IL-18, and IL-1β (P = 0.02, P = 0.007/0.009, P = 0.0001) (Fig. 5e–g).

### NRF-1 is temporally active in regulating pyroptosis-associated signaling in HF

**Figure 6:**
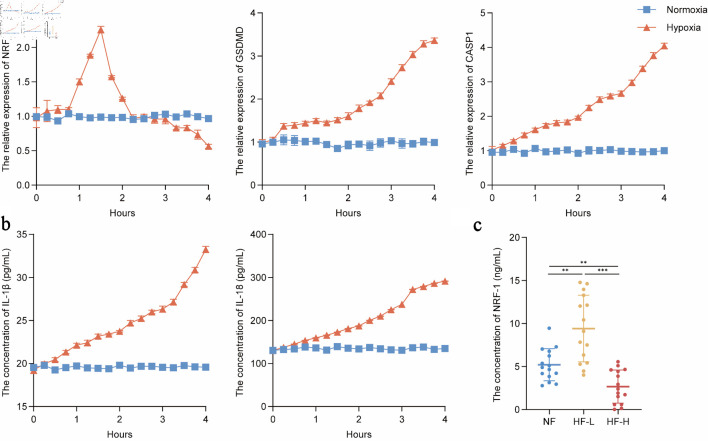
NRF-1 is temporally active in regulating pyroptosis in heart failure. (a) RNA level expression of NRF-1, GSDMD and CASP1 in H9C2 cells during 4 h of hypoxia (n = 3). (b) IL-18 and IL-1β expression in cell culture supernatants in H9C2 cells during 4 h of hypoxia (n = 3). (c) Serum NRF-1 expression in patients of HF-H (n = 15), HF-L (n = 15) and NF (n = 15). **P < 0.01, ***P < 0.001. Patients in New York Heart Association (NYHA) functional class I were classified as HF-L (HF-low), whereas those in class IV were classified as HF-H (HF-high). CASP1: caspase 1; GSDMD: gasdermin D; NRF-1: nuclear respiratory factor-1; HF: heart failure; NF: normal cardiac function.

Although our preliminary findings confirmed the inhibitory role of NRF-1 in pyroptosis-associated signaling during HF, the inconsistent expression of NRF-1 observed across certain samples may be attributed to patients being at different stages of HF at the time of sample collection. To test this hypothesis, we subjected H9C2 cells to hypoxic stimulation and measured the expression of NRF-1 and key pyroptosis-related molecules at 15-min intervals. The results showed that NRF-1 expression began to increase significantly at approximately 1 h, peaked around 2 h to levels comparable to those under normoxia, and then gradually declined over time. In contrast, the expression levels of pyroptosis-related molecules, including GSDMD, caspase-1, IL-18, and IL-1β, exhibited a continuous upward trend. However, during the phase when NRF-1 expression was elevated, the increase in these pyroptosis markers was noticeably attenuated. Once NRF-1 expression returned to baseline levels, the upward trend in pyroptosis-related molecules markedly accelerated (P = 0.005/0.009, P = 0.0001) (Fig. 6a, b). Subsequently, we collected serum samples from patients with HF, classifying those in NYHA functional class I as HF-L (HF-low) and those in class IV as HF-H (HF-high). Serum NRF-1 levels were then measured. The results revealed that NRF-1 expression in the HF-L group was significantly higher than in both the NF and HF-H groups, while HF-H patients exhibited significantly lower NRF-1 levels compared with both the NF and HF-L groups (Fig. 6c).

## Discussion

Pyroptosis involves both canonical and non-canonical pathways. The canonical pathway depends on the activation of caspase-1, which cleaves GSDMD and promotes the release of IL-1β and IL-18, whereas the non-canonical pathway is mediated by caspase-4/5/11 activation [25, 26]. Current research suggests that the canonical pyroptotic pathway contributes to the progression of HF [27–29]. Inflammasome activation in response to myocardial injury triggers pyroptosis and the release of pro-inflammatory cytokines, thereby impairing myocardial repair and exacerbating HF [30, 31]. Consistently, our study also demonstrated significantly elevated expression of pyroptosis-associated signaling in HF patients, and inhibition of pyroptosis in H9C2 cells alleviated hypoxia- and DOX-induced cardiomyocyte injury. In HF models, pyroptosis is markedly upregulated, while NRF-1 effectively inhibits the expression of pyroptosis-related core molecules, thereby suppressing pyroptosis.

NRF is considered to exert protective effects against myocardial injury. In a study investigating the molecular mechanisms mediated by peroxisome proliferator-activated receptor γ coactivator 1α (PGC-1α) and its implications in HF, it was noted that mitochondrial biogenesis is impaired in HF patients, leading to mitochondrial dysfunction [32]. PGC-1α, a key transcriptional regulator of mitochondrial biogenesis, activates the transcription of NRF-1, thereby mediating mitochondrial replication, maintenance, and the production of components of the electron transport chain [33]. The expression of PGC-1α and its downstream molecule NRF is crucial for maintaining NF. As for NRF-1, alpha-lipoic acid activates the NRF-1/ FUN14 domain containing 1 (FUNDC1) pathway to protect against angiotensin II-induced cardiac hypertrophy [34]. However, the specific regulatory role of NRF-1 in HF remains unclear. Recent mechanistic studies have demonstrated that NRF-1 is a stress-responsive transcription factor transiently induced under hypoxic or ischemic conditions, directly regulating mitochondrial quality control and inflammatory signaling through defined downstream pathways [14]. In contrast, our study primarily reveals associative relationships between NRF-1 expression and pyroptosis-related markers, without direct evidence of transcriptional regulation. Our findings demonstrate that NRF-1 expression varies across different stages of HF classified by the NYHA functional grading system, and NRF-1 effectively protects cardiomyocytes under HF conditions. Sustained hypoxic stress eventually overwhelms the protective effects of transient NRF-1 upregulation. Once a critical threshold is reached, namely, when NRF-1 expression begins to decline from its peak, the HF model progresses toward rapid pyroptosis. In addition, we note that NRF-1 expression patterns across publicly available GEO datasets appear inconsistent. While differences in disease stage may plausibly contribute to these discrepancies, other factors such as cohort heterogeneity, sample processing, and technical platform variation may also play a role. Therefore, these observations should be interpreted with caution, and our current results should be considered preliminary.

Previous studies have suggested no direct association between NRF and pyroptosis [21, 22, 24]. Some reports have demonstrated the protective role of NRF-1 in apoptosis. In our study, we found that NRF-1 can suppress hypoxia and DOX-induced pyroptosis in the context of HF. The regulatory role of NRF-1 in pyroptosis during HF is time-dependent and dynamically modulated throughout disease progression. This finding, to some extent, links the NRF family to both HF and pyroptosis.

Nevertheless, our study has several limitations. For instance, while we observed an inverse association between NRF-1 and pyroptosis-related markers, the precise molecular mechanisms remain unclear. At present, it is not established whether NRF-1 directly regulates GSDMD or caspase-1 transcription, or whether its effects occur indirectly through upstream pathways. Future studies employing promoter-binding assays and rescue experiments will be required to clarify whether NRF-1 directly modulates this pathway. In addition, the number of clinical samples analyzed remains limited, and the cohort included only NYHA class I and IV patients, excluding intermediate stages. This selective sampling restricts the generalizability of the proposed dynamic NRF-1 expression model across the full spectrum of HF progression. The unreported clinical variables may influence inflammatory biomarkers. Furthermore, the exclusive use of H9C2 cells limits the translational relevance of our *in vitro* findings, as these rat cardiomyoblasts do not fully recapitulate the complexity of human cardiomyocytes or the *in vivo* cardiac environment. In future studies, larger and well-characterized patient cohorts spanning all NYHA classes, together with mechanistic assays and multiple models, will be needed to fully explore baseline differences and their potential impact on NRF-1 expression.

In conclusion, our findings indicate that NRF-1 may alleviate HF, potentially through the suppression of cellular pyroptosis.

## Data Availability

All datasets involved are included in this manuscript, and the corresponding author can be consulted for more detailed requirements.

## Abbreviations

BCA: bicinchoninic acid
BF: bright field
CASP1: caspase 1
Ctrl: control
DOX: doxorubicin
ECL: enhanced chemiluminescence
ELISA: enzyme-linked immunosorbent assay
FUNDC1: FUN14 domain containing 1
GSDMD: gasdermin D, HF, heart failure
HRP: horseradish peroxidase
IgG: immunoglobulin G
IL: interleukin
LAD: left anterior descending
LVEF: left ventricular ejection fraction
LVIDd: left ventricular internal dimension at end-diastole
LVFS: left ventricular fractional shortening
NF: normal cardiac function
NRF: nuclear respiratory factor
NT-proBNP: N-terminal pro-B-type natriuretic peptide
NYHA: New York Heart Association
PBS: phosphate buffered saline
PGC-1α: peroxisome proliferator-activated receptor γ coactivator 1α
qPCR: quantitative real-time polymerase chain reaction
RIPA: radioimmunoprecipitation assay
SDS: sodium dodecyl sulfate
TBST: Tris buffered saline with Tween
